# Iloprost in COVID-19: The Rationale of Therapeutic Benefit

**DOI:** 10.3389/fcvm.2021.649499

**Published:** 2021-04-23

**Authors:** Paola Maria Faggioli, Nicola Mumoli, Antonino Mazzone

**Affiliations:** ^1^Internal Medicine Azienda Socio Sanitaria Territoriale OVEST Milanese, Legnano Hospital, Milan, Italy; ^2^Internal Medicine Azienda Socio Sanitaria Territoriale OVEST Milanese, Magenta Hospital, Milan, Italy

**Keywords:** COVID-19, iloprost, therapy, thrombosis, inflammation

## Introduction

Recently Moezinia et al. (1) reported a beneficial effect of iloprost in the treatment of acute digital peripheral ischemia that occurred in three patients affected by acute SARS-CoV2 COVID-19 infection, and Johansson et al. (2) reported a benefit of iloprost infusion in ventilated COVID-19 patients.

In both these series, iloprost was infused for five continuous days at the dose of 0.5 mg/kg/min, with a clinical improvement in digital ischemia and respiratory parameters.

Iloprost is a synthetic prostacyclin receptor agonist, used in the treatment of pulmonary arterial hypertension (inhalation), acute and chronic peripheral vascular disease (intravenous) such as critical ischemia, arterial obliterans diseases, digital ulcers, and severe digital ischemia in systemic sclerosis (3–5). Many reports confirm the antithrombotic, anti-inflammatory, and antifibrotic effects of iloprost and highlight its indications not only in the treatment of peripheral vascular diseases but also in critical patients without serious adverse reaction occurence (6).

## Possible Mechanism of Action of Iloprost in COVID-19 Infection

We would like to highlight the mechanisms of action of iloprost in order to clarify its role in inflammation, vasculitis, and thromboembolism in SARS-CoV2 COVID-19 infection. This is because, in agreement with numerous recent studies—also published by our own group (7, 8)—SARS-CoV2 disease showed a clinical and pathophysiological picture very similar to inflammatory diseases with multi systemic involvement and organ damage similar to vasculitis and also similar to microangiopathic and thrombotic damage created by a pro-angiogenic microenvironment (7, 8). It is known that inflammatory storm, by release of cytokines (in particular IL-6), leads to an increased number of circulating activated monocytes/macrophages with the overexpression of adhesion molecules such as integrin complex. The development of cytokine storm induces extensive lung damage in which cells of the inflammatory cascade play a fundamental role as known in viral and bacterial infections. Previous reports supposed that virus such as HIV and SARS-CoV2 can induce variations of serum levels of biomarkers of endothelial damage such as intercellular adhesion molecule 1 (ICAM-1), vascular cell adhesion molecule 1 (VCAM-1), and E-selectin (9). It is also well-known that in COVID-19 infection, serum levels of fractalkine, VCAM-1, ICAM-1, and vascular adhesion protein-1 (VAP-1) were elevated in patients with mild disease, dramatically elevated in severe cases, and decreased in the convalescence phase in correlation with disease course (10–12).

## Discussion

Iloprost, a synthetic prostacyclin analog with potent anti-aggregating and vasodilatory properties, has multiple pharmacological activities such as inhibition of platelet aggregation and leukocyte activation, down-regulation of adhesion molecule expression, improvement of endothelial function, and modulation of the inflammatory status related to systemic atherosclerotic processes (9).

Previous observations documented that CD11b/CD18, a surface receptor expressed in cellular inflammatory responses by neutrophils and activated monocytes macrophages (8, 9), can promote their adhesion to endothelial lining and binding soluble clotting factor X and that fibrinogen acts as a trigger of coagulation cascade and thrombosis, similar to what is reported in ischemic diseases. Also, interleukin-6 (IL-6) can enhance the expression of CD11/CD18 and promote the adhesion of inflammatory monocytes to endothelium mediated by ICAM and their interaction with platelets that could promote a prothrombotic state (8–10).

In effect, inflammation and thrombotic events are frequently observed in the lungs of patients affected by COVID-19 as reported by Ackermann et al. (10) with a prevalence of thrombotic microangiopathy, severe endothelial damage, and intussusceptive neoangiogenesis (8–10).

Furthermore, iloprost is active in the modulation of every mechanism involved in inflammatory response and in systemic damage (8). Although not conclusive, these data seem to confirm the use of iloprost as an additional, safe, and effective therapeutic alternative that deserves to be systematically considered in patients affected by COVID-19.

**Figure 1:**
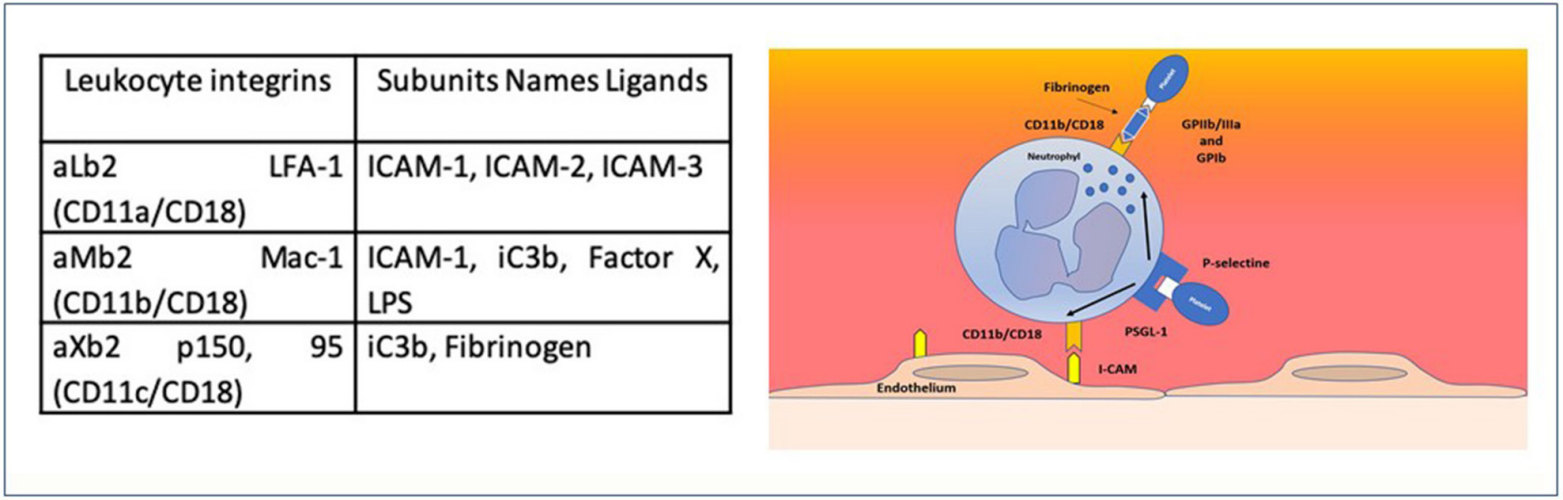
Families of adhesion receptors and their counter receptors. CD11b/CD18 expressed on activated neutrophils supports the interaction between platelets GPIIB/IIIA and GPIb (*via* fibrinogen) and with endothelium *via* ICAM1.

Neutrophils and monocytes/macrophages actively participate in thrombosis and hemostasis cascade, but their role has not yet been sufficiently explored in COVID-19 infection. In COVID-19 pneumonia, persistent activation of circulating neutrophils and monocytes/macrophages, induced by the release of cytokines, in addition to IL-6, has been recently hypothesized (11). Previous observations documented that inflammation mediated by phagocytes in rat lungs is blocked by preincubation with anti-Mo1 monoclonal antibodies (heterodimeric glycoproteins expressed on the plasma membrane of neutrophils, monocytes, macrophages, and a subset of large granular lymphoid cells) preventing pulmonary injury. These antibodies react with CD11b/CD18 integrin complex, which represents a major adhesion molecular structure on neutrophils and monocytes/macrophages. In order to clarify our assumption, we report the ligands of CD11/CD18 integrin complex with molecular adhesion target in Figure 1 and the interaction between CD11b/CD18 expressed on activated neutrophil platelets and endothelium borrowed from our previous papers (12–15) (Figure 1).

It is also well known that iloprost and other synthetic prostanoids have effect *in vivo* and *in vitro* in reducing PMN adhesion; in modulating the surface expression of ICAM-1, VCAM-1, and E-selectin in inflammatory endothelial cells (13); as well as in modulating the expression of CD11b/CD18 in activated macrophages.

Therefore, it can be hypothesized that the therapeutic effects of iloprost in the treatment of vasculopathy induced by COVID-19 can be explained in the modulation of the expression of adhesion molecules and of the interaction between macrophages and activated endothelium.

In support of this hypothesis, in our previous report, we reported a decrease in the serum levels of adhesion molecules, in the expression of CD11b/CD18 in correlation with the trend of COVID-19 infection (6, 14) and related clinical findings.

However, larger studies are needed to confirm these encouraging data observed in this small case series.

## Author Contributions

All authors listed have made substantial, direct and intellectual contribution to the work, and approved it for publication.

## Conflict of Interest

The authors declare that the research was conducted in the absence of any commercial or financial relationships that could be construed as a potential conflict of interest.
